# The relationship between learning styles and academic performance in TURKISH physiotherapy students

**DOI:** 10.1186/s12909-018-1400-2

**Published:** 2018-12-04

**Authors:** Nursen İlçin, Murat Tomruk, Sevgi Sevi Yeşilyaprak, Didem Karadibak, Sema Savcı

**Affiliations:** School of Physical Therapy and Rehabilitation, Dokuz Eylul University, 35340, Inciralti, Izmir, Turkey

**Keywords:** Learning style, Academic performance, Physiotherapy, Student, Turkish

## Abstract

**Background:**

Learning style refers to the unique ways an individual processes and retains new information and skills. In this study, we aimed to identify the learning styles of Turkish physiotherapy students and investigate the relationship between academic performance and learning style subscale scores in order to determine whether the learning styles of physiotherapy students could influence academic performance.

**Methods:**

The learning styles of 184 physiotherapy students were determined using the Grasha-Riechmann Student Learning Style Scales. Cumulative grade point average was accepted as a measure of academic performance. The Kruskal-Wallis test was conducted to compare academic performance among the six learning style groups (Independent, Dependent, Competitive, Collaborative, Avoidant, and Participant).

**Results:**

The most common learning style was Collaborative (34.8%). Academic performance was negatively correlated with Avoidant score (*p* < 0.001, *r* = − 0.317) and positively correlated with Participant score (*p* < 0.001, *r* = 0.400). The academic performance of the Participant learning style group was significantly higher than that of all the other groups (*p* < 0.003).

**Conclusions:**

Although Turkish physiotherapy students most commonly exhibited a Collaborative learning style, the Participant learning style was associated with significantly higher academic performance. Teaching strategies that encourage more participant-style learning may be effective in increasing academic performance among Turkish physiotherapy students.

## Background

Learning can be defined as permanent changes in behavior induced by life [1]. According to experiential learning theory, learning is “the process whereby knowledge is created through the transformation of experience” [2, 3].

Facilitating the learning process is the primary aim of teaching [4]. Understanding the learning behavior of students is considered to be a part of this process [5]. Therefore, the concept of learning styles has become a popular topic in recent literature, with many theories about learning styles put forward to better understand the dynamic process of learning [2, 3].

Learning style refers to an individual’s preferred way of processing new information for efficient learning [6]. Rita Dunn described the concept of learning style as “a unique way developed by students when he/she was learning new and difficult knowledge” [7]. Learning style is about how students learn rather than what they learn [1]. The learning process is different for each individual; even in the same educational environment, learning does not occur in all students at the same level and quality [8]. Research has shown that individuals exhibit different approaches in the learning process and a single strategy or approach was unable to provide optimal learning conditions for all individuals [9]. This may be related to students’ different backgrounds, strengths, weaknesses, interests, ambitions, levels of motivation, and approaches to studying [10]. To improve undergraduate education, educators should become more aware of these diverse approaches [11]. Learning styles may be useful to help students and educators understand how to improve the way they learn and teach, respectively.

Determining students’ learning styles provides information about their specific preferences. Understanding learning styles can make it easier to create, modify, and develop more efficient curriculum and educational programs. It can also encourage students’ participation in these programs and motivate them to gain professional knowledge [9]. Therefore, determining learning style is quite valuable in order to achieve more effective learning. Researching learning styles provides data on how students learn and find answers to questions [5].

Considering the potential problems encountered in the undergraduate education of physiotherapists, determining the learning style of physiotherapy students may enable the development of strategies to improve the learning process [12]. Studies on learning styles in the field of physiotherapy have mostly been conducted in developed countries such as Canada and Australia [13, 14]. A study conducted in Australia examined the learning styles of physiotherapy, occupational therapy, and speech pathology students. The results of this study suggest that optimal learning environment should also be taken into consideration while researching how students learn. The authors also stated that future research was needed to investigate correlations between learning styles, instructional methods, and the academic performance of students in the health professions [14].

To the best of our knowledge, there are no prior publications in the literature that report Turkish physiotherapy students’ learning styles. Furthermore, previous studies mostly used Kolb’s Learning Style Inventory (LSI), Marshall & Merritts’ LSI, or Honey & Mumford’s Learning Style Questionnaire (LSQ) to assess learning styles [5, 13, 15–18]. Some of these studies also suggested that learning behavior and styles should be investigated using different inventories [5]. Moreover, a scale that was indicated as valid and reliable for Turkish population was needed to accurately determine the learning styles of Turkish physiotherapy students. Therefore, we opted to use the Grascha-Riechmann Learning Style Scales (GRLSS) to assess the learning styles of physiotherapy students, which will be a first in the literature.

Learning style preferences are influential in learning and academic achievement, and may explain how students learn [19]. Previous studies have demonstrated a close association between learning style and academic performance [20, 21]. Learning styles have been identified as predictors of academic performance and guides for curriculum design. The aim of this study was to determine whether learning style preferences of physiotherapy students could affect academic performance by identifying the learning styles of Turkish physiotherapy students and assessing the relationship between these learning styles and the students’ academic performance. Since physiotherapy education mainly consists of practice lessons and clinical practice and mostly requires active student participation, we hypothesized that physiotherapy students with a Collaborative learning style according to the GRLSS would have higher academic performance.

## Methods

A cross-sectional survey design using a convenience sample was used. The study population consisted of 488 physiotherapy students who were officially registered for the 2013–2014 academic year in Dokuz Eylul University (DEU) School of Physical Therapy and Rehabilitation. A minimum sample size of 68 participants was calculated with 95% confidence interval and 80% power by using “Epi Info Statcalc Version 6”. Inclusion criteria were (i) age ≥ 17 years, (ii) official registration in DEU School of Physical Therapy and Rehabilitation for the 2013–2014 academic year, (iii) being a first-, second-, third-, or fourth-year undergraduate student of physiotherapy, (iv) ability to read, write, and understand Turkish, and (v) being willing and able to participate in the study. Exclusion criteria were (i) unwilling to participate in the study, (ii) inability to read, write, and understand Turkish. The questionnaire was distributed to the physiotherapy students in a classroom setting during the final exam week of the academic year. Due to the absence of participants who did not attend final exams and were not actively attending classes (non-attendance students), questionnaires were distributed to 217 students in total.

184 physiotherapy students with a mean ± SD age of 21.52 ± 1.75 years participated in the study. Participants were informed verbally and in writing about the purpose of the study and the survey that would be implemented. A research assistant was available in the classroom to provide assistance if required. Demographic characteristics (age, gender, undergraduate year) comprised the first section of the questionnaire, followed by the GRLSS to assess learning style.

Cumulative grade point average (CGPA) shown on the students’ transcripts was used as the measure of academic performance. The students’ CGPAs at the end of the 2013–2014 academic year were obtained from the records held in the student affairs unit of the DEU School of Physical Therapy and Rehabilitation. CGPA was derived by multiplying the grade point (out of 100) with the credit units for each module or course and then dividing the total sum by the total credit units taken in the program.

The local university ethics committee provided ethical approval and informed consent was obtained from the participants before inclusion. Ethical protocol number was 1432-GOA.

### Data collection

#### Grasha-Riechmann student learning style scales

The GRLSS is a five-point Likert-type scale (*response format: strongly disagree, moderately disagree, undecided, moderately agree, strongly agree*) consisting of 60 items which was designed based on student interviews and survey data [22, 23]. In accordance with the response to student attitudes toward learning, classroom activities, teachers and peers, six learning styles were defined [24]. Learning styles that form subscales are the Independent, Avoidant, Collaborative, Dependent, Competitive, and Participant learning styles [24, 25]. The six main styles in the GRLSS are described in Table 1 and the scoring of the GRLSS is shown in Table 2 [23, 24]. The GRLSS was adapted to Turkish in 2003 and found to have good reliability [25] (Table 3).

**Table 1:** Description of the six main style in the Grasha-Riechmann Student Learning Style Scales

Independent	students prefer self-pace instruction and prefer to study alone rather than with other students. They like to think for themselves and are confident in their abilities. They like maximum choice and flexibility and minimum of structure and form. They prefer independent assignments and self-paced instruction.
Dependent	students prefer that the teacher guides them and tells them what to do. They only learn what is required and they look up to the teacher for specific guidelines on what to do. They show little intellectual curiosity. They prefer outlines, clear instructions and guidelines and teacher-centered classroom activities.
Competitive	students learn in order to perform better than their peers. They feel that they have to compete with other students in the class to get a grade. They like to be the center of attention and to receive recognition for their academic achievements.
Collaborative	learners learn by sharing and cooperating with their teachers and peers. They prefer lectures with small group discussions and group projects.
Avoidant	learners are not enthusiastic about attending class or acquiring class content. They don’t like to participate in class activities and are sometimes overwhelmed by class activities.
Participant	learners are interested in class activities and discussions. They enjoy coming to class and participating in class activities. They like opportunities to discuss class material and readings.

**Table 2:** Scoring of the Grasha-Riechmann Student Learning Style Scales

Independent		Avoidant		Collaborative		Dependent		Competitive		Participant	
01.	…..	02.	…..	03.	…..	04.	…..	05.	…..	06.	…..
07.	…..	08.	…..	09.	…..	10.	…..	11.	…..	12.	…..
13.	…..	14.	…..	15.	…..	16.	…..	17.	…..	18.	…..
19.	…..	20.	…..	21.	…..	22.	…..	23.	…..	24.	…..
25.	…..	26.	…..	27.	…..	28.	…..	29.	…..	30.	…..
31.	…..	32.	…..	33.	…..	34.	…..	35.	…..	36.	…..
37.	…..	38.	…..	39.	…..	40.	…..	41.	…..	42.	…..
43.	…..	44.	…..	45.	…..	46.	…..	47.	…..	48.	…..
49.	…..	50.	…..	51.	…..	52.	…..	53.	…..	54.	…..
55.	…..	56.	…..	57.	…..	58.	…..	59.	…..	60.	…..
Total:	…..	Total:	…..	Total:	…..	Total:	…..	Total:	…..	Total:	…..
Mean:	…..	Mean:	…..	Mean:	…..	Mean:	…..	Mean:	…..	Mean:	…..

The numbers below represent the items in the questionnaire that correspond to each of the learning style dimensions on the questionnaire. To score this questionnaire, place the ratings assigned to each item in the space provided. Sum each column and divide by 10 to obtain the mean score for each scale

**Table 3:** Cronbach Alpha reliability coefficients of the Turkish version of Grasha-Riechmann Student Learning Style Scales

	Number of students	Number of items	Alpha
Competitive	184	10	,7951
Dependent	184	10	,6005
Collaborative	184	10	,7514
Avoidant	184	10	,6831
Independent	184	10	,6774
Participant	184	10	,7578
TOTAL	184	60	,8319

The learning styles of the physiotherapy students in the current study were identified according to GRLSS and the students were grouped based on their predominant (highest scoring) style. The mean and median academic performance values of each group were calculated and the significance of the differences between groups was statistically analyzed.

#### Statistical analysis

Statistical analyses were performed to compare academic performances among the learning style groups and test the significance of pairwise differences. All data were analyzed using Statistical Package for Social Science software (IBM Corporation, version 20.0 for Windows). Descriptive statistics were summarized as frequencies and percentages for categorical variables. Continuous variables were presented as mean and standard deviation when normally distributed and as median and interquartile range when not normally distributed. Mann-Whitney U test was used for between-group analyses of abnormally distributed variables. The variables were investigated using visual (histograms, probability plots) and analytical methods (Kolmogorov-Smirnov/Shapiro-Wilk test) to determine whether they showed normal distribution. As parameters were not normally distributed, the correlation coefficients and their significance were calculated using Spearman test. Strength of correlation was defined as very weak for r values between 0.00–0.19, weak for r values between 0.20–0.39, moderate for r values between 0.40–0.69, strong for r values between 0.70–0.89, and very strong for r values over 0.90 [26]. As the academic performance was not normally distributed, the Kruskal-Wallis test was conducted to compare this parameter among the six learning style groups. The Mann-Whitney U Test was performed to test the significance of pairwise differences using Bonferroni correction to adjust for multiple comparisons. An overall 5% type-I error level was used to infer statistical significance (*p* < 0.05).

## Results

A total of 217 physiotherapy students were invited to participate in the study. Eighteen students refused to participate. Fifteen surveys were discarded due to missing item responses. As a result, data obtained from 184 students were used for the analyses. Overall response rate was 84.8%.

Demographic characteristics (gender, year) and learning style preferences are presented in Table 4. The most common learning styles among the physiotherapy students according to the GRLSS were Collaborative (34.8%) and Independent (22.3%). The results of GRLSS subscale scores were given in Table 5. The highest subscale score was Collaborative (Mean ± SD = 3.57 ± 0.62), while Competitive score was the lowest (Mean ± SD = 2.81 ± 0.69).

**Table 4:** Demographic characteristics and learning style preferences of the surveyed physiotherapy students

*n* = 184	Number (n)	Percent (%)
Gender	78	42.4
Female	106	57.6
Male	46	25.0
Grade		
1st		
2nd	60	32.6
3rd	35	19.0
4th	43	23.4
Learning Style		
Independent	41	22.3
Avoidant	27	14.7
Collaborative	64	34.8
Dependent	25	13.6
Competitive	10	5.4
Participant	17	9.2

**Table 5:** GRLSS sub-scale scores of the physiotherapy students

*n* = 184	Mean ± SD	Minimum	Maximum
Independent score	3.56 ± 0.55	1.70	5.00
Avoidant score	3.16 ± 0.59	1.60	4.80
Collaborative score	3.57 ± 0.62	1.00	4.90
Dependent score	3.50 ± 0.44	2.10	4.70
Competitive score	2.81 ± 0.69	1.00	4.60
Participant score	3.15 ± 0.71	1.00	4.80

A moderate positive correlation between academic performance and Participant score was found *(p < 0.001, r = 0.400)*. A weak negative correlation was also found between academic performance and Avoidant score *(p < 0.001, r = − 0.317)*. No other significant correlation between academic performance and subscale scores was found (Table 6)*.*

**Table 6:** Correlation matrix among variables

	Independent score	Avoidant score	Collaborative score	Dependent score	Competitive score	Participant score
Academic Performance	*r* = 0.109 *p* = 0.142	*r* = −0.317* p = 0.000*	*r* = 0.051 *p* = 0.489	*r* = 0.053 *p* = 0.478	*r* = 0.105 *p* = 0.155	*r* = 0.400* p= 0.000*

*Spearman Correlation Analysis: *p* < 0.05

When students were grouped according to learning styles, between-group (Kruskal-Wallis) analysis showed a significant difference in the academic performance of the groups *(p < 0.001).* Post-hoc (Mann-Whitney U) analysis revealed significantly higher academic performance in the Participant learning style group compared to all of the other learning style groups (Independent, Avoidant, Collaborative, Dependent, and Competitive) (Table 7).

**Table 7:** Comparison of academic performances among the learning style groups

	Independent (Group 1) Median (min-max)	Avoidant (Group 2) Median (min-max)	Collaborative (Group 3) Median (min-max)	Dependent (Group 4) Median (min-max)	Competitive (Group 5) Median (min-max)	Participant (Group 6) Median (min-max)	Kruskal-Wallis *p*-value	Comparison group	Post-hoc *p*-value
Academic Performance	72.39 (62.66–83.13)	72.13 (61.49–82.14)	71.30 (58.11–90.61)	74.18 (50.38–93.69)	70.57 (18.38–76.14)	79.77 (63.97–89.31)	0.000*	1 vs 2 1 vs 3 1 vs 4 1 vs 5 1 vs 6 2 vs 3 2 vs 4 2 vs 5 2 vs 6 3 vs 4 3 vs 5 3 vs 6 4 vs 5 4 vs 6 5 vs 6	0.661 0.438 0.318 0.265 0.000** 0.835 0.190 0.516 0.000** 0.121 0.492 0.000** 0.080 0.001** 0.000**

Kruskal-Wallis Test: *p < 0.05

Post-hoc (Mann-Whitney U) test: **p < 0.05/15 = **p < 0.003 (with Bonferroni correction)

## Discussion

The current study assessed the learning styles of Turkish physiotherapy students, and investigated the relationship between their learning styles and academic performance. The results revealed that the Collaborative learning style was most common among the Turkish physiotherapy students. However, students with Participant learning style had statistically higher academic performance when compared to the others. In addition, we found a positive correlation between Participant score and academic performance of the students, which supports the previous finding, while a negative correlation was found between Avoidant score and academic performance. In the case of physiotherapy students in this study, the emphasis should be on developing Participant and Collaborative learning skills. This might involve providing more class activities, discussions, and group projects.

The physiotherapy program at DEU has a combined case study-based and traditional style curriculum including lectures, tutorials, seminars, case study presentations, and supervised small group clinical practice in the hospital and at other health centers. Learning tasks and assessment methods include individual written examinations, practical examinations, homework and assignments as well as collaborative oral presentation and research projects. In the physiotherapy discipline, clinical practice improves students’ occupational skills and is seen as a crucial part of the teaching process [12, 27]. Similarly, the teaching and learning approach at DEU is heavily based on practical training and requires active participation and group work. This could be a reason for the greater preference for Collaborative learning style.

Previous studies have indicated that physiotherapy students prefer abstract learning styles [28] and have desirable approaches to studying [29]. Canadian and American physiotherapy students preferred Converger (40 and 37% respectively) or Assimilator (35 and 28% respectively) learning styles [13]. According to descriptions of the learning style categories in the Kolb LSI, Convergers enjoy learning through activities like homework problems, computer simulations, field trips, and reports and demonstrations presented by others. On the other hand, Assimilators prefer attending lectures, reading textbooks, doing independent research and watching demonstrations by instructors when learning. In our study, Turkish physiotherapy students preferred Collaborative (34.8%) or Independent (22.3%) learning styles. According to GRLSS, Collaboratives prefer lectures with small group discussions and group projects (similar to Assimilators), while Independents prefer self-pace instruction and studying alone (similar to Convergers). Therefore, it can be concluded that learning styles of Canadian, American, and Turkish physiotherapy students are similar to each other.

Katz and Heimann used the Kolb LSI in their study and reported average learning style scores instead of the number of students in each of the four learning styles. They reported Converger as the “average” learning style for physiotherapy students [30]. In our study, the largest proportion of the physiotherapy students had a Collaborative learning style. Moreover, the average learning style was also Collaborative, with the highest average score.

Competitive learning style was the least frequently preferred (5.4%) by Turkish physiotherapy students in our study. The low preference for Competitive learning style indicates that students were less likely to compete with other students in the class to get a grade. Mountford et al. assessed learning styles of Australian physiotherapy students using Honey & Mumford’s LSQ and found that the Pragmatic learning style was the least preferred. According to LSQ, Pragmatists tend to see problem solving as a chance to rise to a challenge [31]. Considering that both Competitives and Pragmatists like challenges, the least frequently preferred styles of Australian and Turkish physiotherapy students seem to be similar to each other.

Alsop and Ryan pointed out that “personal awareness of learning styles and confidence in communicating this are first steps to achieve an optimal learning environment” [32]. According to Kolb’s theory, a preferred learning style affects a person’s problem solving ability [13]. Wessel et al. also stated that in order to provide students the best learning opportunity, educators must be aware of the learning styles and students’ ability to solve problems [13]. Indeed, evidence supporting these views can be found in the literature. Previous studies showed that students who were aware of their learning style had improved academic performance [33, 34]. Nelson et al. found that college students who were tested on their learning style and were given appropriate education according to their learning style profile achieved higher academic performance than other students [33]. Linares also investigated learning styles in different health care professions (physiotherapy, occupational therapy, physician assistants, nursing and medical technology) and found a significant relationship between learning style and students’ readiness to undertake self-directed learning [15]. However, Hess et al. found no association between learning style and problem-solving ability in their study [35].

While planning this study, we hypothesized that students with a Collaborative learning style would have higher academic performance. Although the Collaborative learning style was the most common, these students did not show significantly higher academic performance. However, students with Participant learning style had statistically higher academic performance when compared to the other learning style groups. Characteristics specific to the Participant learning style are enjoyment from attending and participating in class and interest in class activities and discussions. These students enjoy opportunities to discuss class materials and readings. This may suggest that increasing in-class activities and discussions, which encourage participant-style learning, is needed to increase academic performance. Another approach would be to adapt teaching strategies according to the characteristics of Collaboratives, as they represented the largest body of students. Creating a convenient environment in which students could spend more time sharing and cooperating with their teacher and peers may facilitate collaborative learning, thus enhancing academic performance. Organizing the curriculum to include small group discussions within lectures and incorporate group projects may also be beneficial. As Ford et al. stated, “*Identification teaching profiles could be used to tailor the collaborative structure and content delivery*” [36].

The most important reason for determining learning style is to create a proper teaching strategy [37–40]. However, there seems to be no exact relationship between students’ learning style and the curriculum of a program described in the literature [13]. Learning style alone is not the only factor that may influence a learning situation. Many factors (educational and cultural context of university, individual awareness, life experience, other learning skills, effect of educator, motivation, etc.) may influence the learning process [31]. Therefore, expecting a simple relationship between learning style and teaching strategy may not be realistic. Moreover, the review of Pashler et al. showed that there was virtually no evidence that people learn better when teaching style is tailored to match students’ preferred learning style [41]. Nevertheless, future studies investigating physiotherapy educators’ teaching styles and their association with learning styles and academic performance may elucidate this complex issue.

The major strength of this study is that, to the best of our knowledge, ours is the first study investigating the learning styles of Turkish physiotherapy students with relation to academic performance.

There were some limitations to this study. It should be noted that learning style is a self-reported measure that can change based on experience and the demands of a situation. Therefore, it is subjective and able to provide adaptive behavior [42]. It should also be kept in mind that the conclusions of this study could be limited due to the cross-sectional design, and respondent bias may be an issue because convenience sampling was used to recruit participants. One possible limitation of the study could be the fact that the three of the scale reliabilities reported for GRLSS was poor.

This study investigated the learning styles of physiotherapy students in only one university (DEU) and this could preclude the generalization of our results. Subsequent studies should include students enrolled in the physiotherapy departments of multiple universities in Turkey to achieve an accurate geographical representation. Moreover, future studies on this topic should be conducted in collaboration with universities in Europe, with which we share a cultural connection.

## Conclusions

The results of this study showed that the Collaborative learning style was most common among Turkish physiotherapy students. On the other hand, the physiotherapy students with Participant learning style had significantly higher academic performance than students with other learning styles. Teaching strategies consistent with the unique characteristics of the Participant learning style may be an effective way to increase academic performance of Turkish physiotherapy students. Incorporating more in-class activities and discussions about class material and readings may facilitate Participant learning, thus impacting academic performance positively. Another approach may be to adopt teaching strategies that target the predominant Collaborative learning style. Creating a convenient environment for students to share and cooperate with their teacher and peers and organizing the curriculum to include more small group discussions and group projects may also be supportive. Future studies should investigate physiotherapy educators’ teaching styles and their relations with learning styles and academic performance.

## Abbreviations

CGPA: Cumulative Grade Point Average
DEU: Dokuz Eylul University
GRLSS: Grascha-Riechmann Learning Style Scales
LSI: Learning Style Inventory
LSQ: Learning Style Questionnaire
